# Prevalence of Tick-Borne Pathogens in Questing *Ixodes ricinus* and *Dermacentor reticulatus* Ticks Collected from Recreational Areas in Northeastern Poland with Analysis of Environmental Factors

**DOI:** 10.3390/pathogens11040468

**Published:** 2022-04-14

**Authors:** Anna Grochowska, Justyna Dunaj-Małyszko, Sławomir Pancewicz, Piotr Czupryna, Robert Milewski, Piotr Majewski, Anna Moniuszko-Malinowska

**Affiliations:** 1 Department of Infectious Diseases and Neuroinfections, Medical University of Białystok, 15-540 Białystok, Poland; dunaj.justyna@wp.pl (J.D.-M.); spancewicz@interia.pl (S.P.); avalon-5@wp.pl (P.C.); anna.moniuszko@umb.edu.pl (A.M.-M.); 2 Department of Statistics and Medical Informatics, Medical University of Białystok, 15-295 Białystok, Poland; robert.milewski@umb.edu.pl; 3 Department of Microbiological Diagnostics and Infectious Immunology, Medical University of Białystok, 15-269 Białystok, Poland; piotr.majewski@umb.edu.pl

**Keywords:** urban, suburban, *Borrelia*, *miyamotoi*, *Babesia*, *Anaplasma*, co-infection

## Abstract

Ticks, such as *Ixodes ricinus* and *Dermacentor reticulatus*, act as vectors for multiple pathogens posing a threat to both human and animal health. As the process of urbanization is progressing, those arachnids are being more commonly encountered in urban surroundings. In total, 1112 *I. ricinus* (*n* = 842) and *D. reticulatus* (*n* = 270) ticks were collected from several sites, including recreational urban parks, located in Augustów and Białystok, Poland. Afterwards, the specimens were examined for the presence of *Borrelia* spp., *Babesia* spp., *Anaplasma phagocytophilum*, *Rickettsia* spp., *Bartonella* spp., and *Coxiella burnetii* using the PCR method. Overall obtained infection rate reached 22.4% (249/1112). In total, 26.7% (225/842) of *I. ricinus* was infected, namely with *Borrelia* spp. (25.2%; 212/842), *Babesia* spp. (2.0%; 17/842), and *A. phagocytophilum* (1.2%; 10/842). Among *D. reticulatus* ticks, 8.9% (24/270) were infected, specifically with *Babesia* spp. (7.0%; 19/270), *A. phagocytophilum* (1.1%; 3/270), and *Borrelia burgdorferi* s.l. (0.7%; 2/270). No specimen tested positively for *Rickettsia* spp., *Bartonella* spp., or *Coxiella burnetii*. Co-infections were detected in 14 specimens. Results obtained in this study confirm that *I. ricinus* and *D. reticulatus* ticks found within the study sites of northeastern Poland are infected with at least three pathogens. Evaluation of the prevalence of pathogens in ticks collected from urban environments provides valuable information, especially in light of the growing number of tick-borne infections in humans and domesticated animals.

## 1. Introduction

Over the past few decades, the phenomenon of urbanization has increased significantly worldwide. Currently, more than 50% of the human population lives in urban areas, and by 2050, this number is expected to rise to 75% [1]. The transformation of wild landscapes into cities and recreational areas causes major changes in the distribution of wildlife. Ticks are an example of arthropods that adapted to the new conditions, increasing the risk of human exposure to tick-borne pathogens. Those arachnids are typically associated with forests, meadows, and other rural landscapes. However, in recent decades, reports of their presence in urban surroundings are becoming increasingly frequent [1,2,3]. The presence of well-known tick-borne pathogens, such as *Borrelia burgdorferi* sensu lato, *Anaplasma phagocytophilum*, or *Babesia* spp., among others, has been detected in ticks collected from recreational areas in multiple studies across Europe [4,5,6,7,8,9,10,11].

Alongside host availability, environmental conditions, such as temperature and humidity, are the most important factors influencing the activity, development, and survival of the ticks [12]. Higher temperatures and humidity contribute to faster development, shorter lifecycle, and increased activity of ticks [13]. However, a warmer climate may contribute to decreased humidity and thus causing higher tick mortality rates [12,13].

Both cities chosen for this study are located in the Podlaskie Voivodeship. This region has one of the highest incidence rates of Lyme disease in Poland, and it is twice as high as the national average [14], hence research regarding ticks occurring within this area is of great importance.

This study aimed to assess the prevalence of six chosen tick-borne pathogens: *Borrelia* spp., *A. phagocytophilum*, *Babesia* spp., *Rickettsia* spp., *Coxiella burnetii*, and *Bartonella* spp. in questing *Ixodes ricinus* and *Dermacentor reticulatus* ticks collected from recreational areas of Białystok and Augustów, Poland, as well as to analyze the influence of the climatic factors on tick infection rates.

## 2. Materials and Methods

### 2.1. Collection of Ticks

Questing *I. ricinus* and *D. reticulatus* ticks were sampled with the usage of the flagging method, from recreational sites within the cities of Białystok (2017–2019) and Augustów (2018–2019), both located in the Podlaskie Voivodeship. During each sampling, air temperature and relative air humidity were measured several times and the average value was documented.

In Białystok, the collection took place in the Zwierzyniecki Forest Nature Reserve (53°6′45″ N, 23°9′41″ E), which is dominated by hornbeam, oak, pine, and birch trees. This area is located approximately 2 km from the city center and in the immediate vicinity of the University of Bialystok campus. It is commonly used for recreational purposes, such as hiking, jogging, dog walking, and biking, among others. The sampling of *I. ricinus* and *D. reticulatus* ticks took place in July and August of 2017 and from April to October in 2018–2019. *D. reticulatus* ticks included in this study were collected in the year 2019, while those from 2018 were analyzed previously [15]. The frequency of tick sampling in Białystok was approximately once a week for up to 3 h, depending on weather conditions.

In Augustów, two sites were chosen for tick collection. The first location was the “Królowa Woda” resort (53°49′27.3″ N, 22°58′41.3″ E), located on Lake Sajno. The second location was the Public Beach (53°51′14.9″ N, 22°59′03.9″ E), located on Necko Lake. Both areas are surrounded by a forest dominated by pine and spruce trees. However, birches, maples, hornbeams, lindens, and alders may also be found. These are popular recreational sites, with designated swimming areas, water equipment rentals, gastronomic premises, and connections to walking and bike paths. The ticks were collected in the spring and autumn of 2018–2019, over several trips for up to 6 h.

Obtained individuals were placed separately in Eppendorf tubes. Later, each one was identified for species and stage [16] and stored at +4 °C for up to 1 week, until further DNA extraction.

### 2.2. DNA Isolation

Collected ticks were crushed individually in a mortar with the addition of 1.5 mL of PBS (without Ca^2+^ and Mg^2+^ ions). Obtained homogenate was centrifuged. Afterwards, 300 µL of supernatant was used to perform DNA extraction (EurX DNA Isolation Kit, Gdańsk, Poland) in accordance with the manufacturer’s instructions. Finally, 100 µL of obtained DNA extracts were stored at −20 °C until further analyses.

### 2.3. PCR Amplification

All PCR reactions were performed on the SensoQuest LabCycler (SensoQuest, Göttingen, Germany). Obtained DNA isolates were pooled by five (15 µL of each). If a pool tested positive, components were tested again, separately, in order to obtain the exact number of infected specimens.

Further PCR and electrophoresis, as well as sequencing analysis for the detection of chosen pathogens, were performed according to the methods previously described by Grochowska et al. [15].

For identification of *Borrelia* spp., a 120-bp fragment of the 16S rRNA gene encoding small ribosomal subunit was amplified. PCR was performed with the *Borrelia burgdorferi* PCR kit (GeneProof, Brno, Czech Republic) for in vitro diagnostics. The reaction program was designed in compatibility with GeneProof instruction with its own modifications and consisted of the following steps: UDG decontamination at 37 °C for 2 min, initial denaturation at 95 °C for 10 min, amplification for 45 cycles (denaturation at 95 °C for 5 s, annealing at 60 °C for 40 s, extension at 72 °C for 20 s), and final extension at 72 °C for 2 min.

For *A. phagocytophilum* DNA detection, a nested PCR, targeting a fragment of 16S rDNA gene encoding small ribosomal 16S RNA subunit, was used. Reactions were performed with the Anaplasma PCR kit (Blirt-DNA Gdańsk, Gdańsk, Poland), according to the manufacturer’s instructions.

Identification of *Babesia* spp. was performed using a fragment of the 18S rDNA gene, encoding a small ribosomal subunit, localized on conservative region V4. PCR was performed with Taq PCR Core Kit (Qiagen, Hilden, Germany) with the use of a pair of highly specific primers (Sigma-Aldrich, Schnelldorf, Germany): 18S rDNA BAB-F2 sense 5′-GAC ACA GGG AGG TAG TGA CAA G-3′ and 18S rDNA BAB-R2 antisense 5′-CTA AGA ATT TCA CCT CTG ACA GT-3′ [17,18,19,20].

For *Rickettsia* spp., *Bartonella* spp., and *C. burnetii* identification, the Vet PCR *RICKETTSIA*, The Hum PCR *BARTONELLA*, and The Hum PCR *Coxiella burnetii* detection kits (BioIngenTech, Concepción, Chile) were used, respectively. All reactions were performed in accordance with manufacturer’s instructions.

Electrophoresis on 2% agarose gel (Sigma-Aldrich, Darmstadt, Germany) stained with ethidium bromide (5 µg/mL; Syngene, Frederick, MD, USA) was used to separate the amplicons, as described by Grochowska et al. [15].

Samples positive for *Borrelia* spp. and *Babesia* spp. were sequenced by Macrogen (Amsterdam, The Netherlands). In total, 5 µL of obtained amplification products were mixed with specific primers: BIG BOR-F1 (5 µL, 50 mM) and BIG BOR-R1 (5 µL, 50 mM) for *Borrelia* spp. and those used previously for PCR for *Babesia* spp. Prepared samples were sent to Macrogen, where they were sequenced from both sides. All positive *A. phagocytophilum* amplicons were purified with the Wizard^®^ SV Gel and PCR Clean-Up System (Promega, Madison, WIS, USA) and subjected to Sanger sequencing at a commercial facility (Macrogen Europe, Maastricht, The Netherlands).

Afterwards, the results were compared with sequences deposited in the GenBank using the BLAST program. Sequences with the highest compatibility were recorded.

### 2.4. Evolutionary Relationships of Taxa

The evolutionary history of the various *Borrelia* and *Babesia* genospecies was inferred by using the Neighbor-Joining method [21]. The evolutionary distances were computed using the Tamura-Nei method [22] were are in the units of the number of base substitutions per site. Evolutionary analyses were conducted in MEGA X [23] with subsequent phylogenetic tree visualization using iTOL v61 [24].

This analysis involved

- 118 nucleotide sequences for *Borrelia* isolated from *I. ricinus* in Białystok (1264 bp),
- 94 nucleotide sequences for *Borrelia* isolated from *I. ricinus* in Augustów (1243 bp),
- 17 nucleotide sequences for *Babesia* isolated from *I. ricinus* (289 bp),
- 19 nucleotide sequences for *Babesia* isolated from *D. reticulatus* (275 bp).

### 2.5. Statistical Analysis of Previous and Present Research

This study is the expansion of the previous study, focusing on *D. reticulatus* ticks collected in Białystok in 2018 [15]. Since the specimens were analyzed for the presence of the same six pathogens and were obtained in the same area, it was decided to combine the results and perform statistical analysis on a larger study group, including all collection years, in order to obtain more accurate results.

Aforementioned research included 368 *D. reticulatus* ticks collected in the Zwierzyniecki Forest Nature Reserve in Białystok, Poland, from April to October 2018. Among those, 9.2% were infected with *Babesia* spp., 0.8% with *A. phagocytophilum*, and 0.3% with *B. burgdorferi* s.l.

Statistical analysis was performed using the Statistica 12.0 program (StatSoft, Tulsa, OK, USA).

The Mann-Whitney test was used to assess the prevalence of pathogens in relation to temperature (above and below 20 °C) and humidity (above and below 80%), both with division to the sampling season (April–July, August–October). Overall infection rate, as well as the prevalence of individual pathogens between the two tick species, and developmental stages were also compared using the same test.

Additionally, logistic regression analysis was performed in order to compare the influence of multiple factors.

Statistical significance was established as *p* < 0.05.

## 3. Results

**Figure 1:**
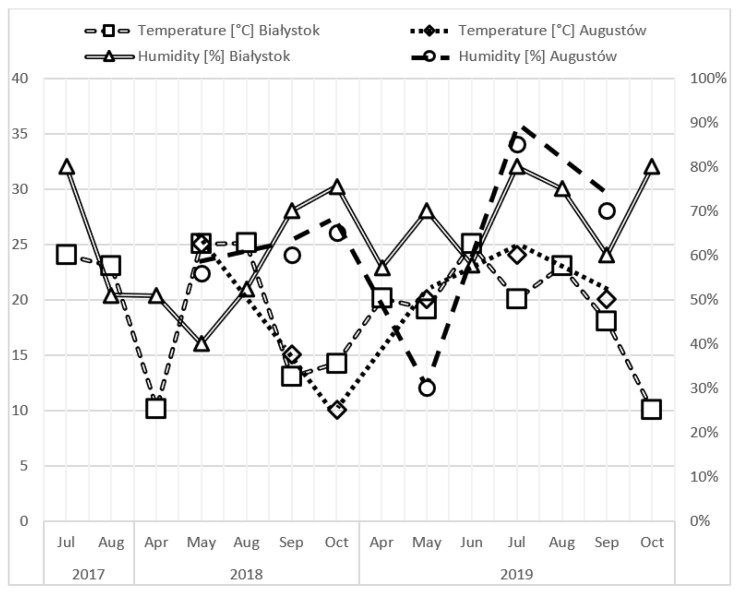
Temperature and humidity recorded during collection of the ticks in Białystok and Augustów.

In total, 1112 ticks were collected from the study areas. The majority of them (842), specifically 460 from Białystok and 382 from Augustów, were classified as *I. ricinus* (239 females, 207 males, 319 nymphs, 77 larvae). The remaining 270 individuals (252 from Białystok and 18 from Augustów) were identified as *D. reticulatus* (162 females, 100 males, 8 nymphs) (Table 1). Environmental conditions (temperature and humidity) recorded during collection of the ticks are presented in Figure 1.

**Figure 2:**
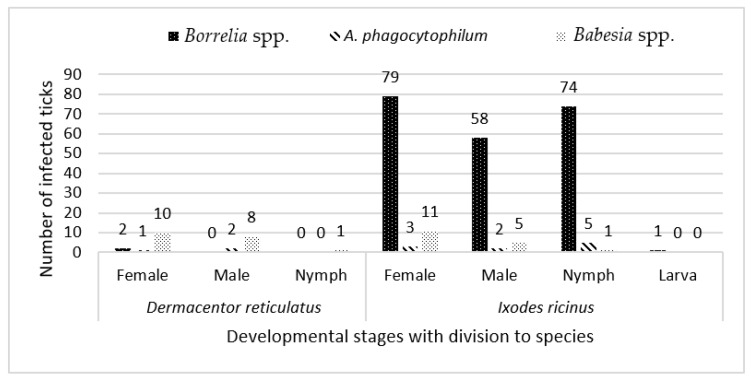
The total number of tick-borne pathogen compositions among different developmental stages of sampled *Dermacentor reticulatus* and *Ixodes ricinus* ticks collected in Białystok and Augustów in years 2017–2019.

**Figure 3:**
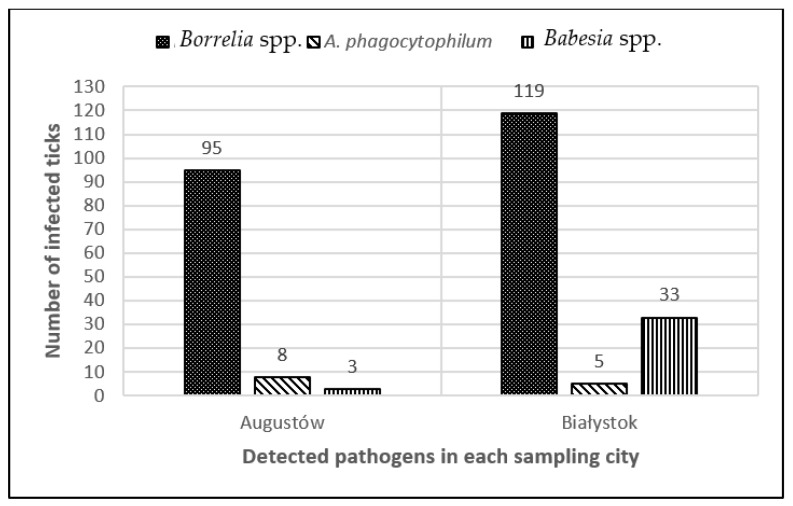
The total number of pathogen compositions with division to the sampling site.

Presence of tick-borne pathogens was confirmed in 22.4% (249/1112) of the ticks. Total infection rate for *I. ricinus* was 26.7% (225/842; 85 females, 60 males, 79 nymphs, 1 larva). The most prevalent pathogen was *Borrelia* spp. (25.2%; 212/842), followed by *Babesia* spp. (2.0%; 17/842) and *A. phagocytophilum* (1.2%; 10/842). Among 270 *D. reticulatus*, 8.9% (24/270) were infected, namely with *Babesia* spp. (7.0%; 19/270), *A. phagocytophilum* (1.1%; 3/270), and *Borrelia* spp. (0.7%; 2/270) (Figure 2). No specimen tested positively for *Rickettsia* spp., *Bartonella* spp., or *C. burnetii* (Table 2). The most prevalent pathogen in both sampling sites was *Borrelia* spp., followed by *Babesia* spp. in Białystok and *A. phagocytophilum* in Augustów (Figure 3).

### 3.1. Sequencing Analysis

In Białystok, among 118 *Borrelia*-positive *I. ricinus* ticks, the majority was identified as *Borrelia afzelii* (65.3%; 77/118) with a similarity ranging from 86.88% to 98.69% to bacteria isolated both from humans in Austria and Germany (GenBank: CP009058.1, CP018262.1) and from ticks in Russia and France (CP009212.1, MW301927.1). Seventeen ticks (14.4%) showed 89.43–98.61% identity to *Borrelia garinii* isolated from different tick species from France, Spain (GenBank: CP028861.1, DQ147793.1), and Russia (GenBank: EF488989.1, KY312011.1, KY312012.1), as well as from human blood in China (GenBank: AY342031.1). Ten sequences (8.4%) were 83.97–98.25% identical to *Borrelia burgdorferi* sensu stricto, found in *I. ricinus* in the United Kingdom (GenBank: X98233.1) and in *Peromyscus leucopus* in the USA (GenBank: CP031412.1). The next 10 sequences (8.4%) were identified as *Borrelia miyamotoi* with a similarity range of 95.04–98.94% (GenBank: CP046389.1; *Ixodes* eggs, Czech Republic), while the remaining four sequences (3.4%) showed 92.49–97.05% identity to *Borrelia lusitaniae* (GenBank: AB091820.1; *I. ricinus*, Turkey). One *Borrelia*-positive *D. reticulatus* tick showed 96.86% similarity to *B. afzelii* (GenBank: CP009058.1). As for *Babesia* spp. sequencing, 15 out of 17 (88.2%) *I. ricinus* ticks showed 87.10–99.47% identity to *Babesia microti* isolated from ticks and small mammals from Turkey, China, Thailand, and Germany (GenBank: MH628094.1, KY649348.1, MG199182.1, MN355504.1, KP055650.1), while two were 91.83% and 99.36% identical to *Babesia venatorum* (GenBank: KR003828.1). Among *Babesia*-positive *D. reticulatus* ticks, 15 (93.8%; 15/16) were identified as *Babesia canis*, with 86.60–98.98% similarity to small and medium mammals from Lithuania, Poland, Serbia, Iran, Ukraine, Turkey, Romania, and Italy (GenBank: MN078319.1, MK872807.1, MH702200.1, MN173223.1, MN704759.1, MK934420.1, MG569903.1, KU821654.1, KT844899.1), as well as ticks from Ukraine and Poland (GenBank: MT346582.1, MF797820.1). One sequence showed 93.69% identity to *B. microti* (GenBank: KP055650.1, *Myodes glareolus*, Germany).

As for samples from Augustów, 34 out of 94 *Borrelia*-positive *I. ricinus* ticks were 82.89–98.25% identical to *B. garinii* (GenBank: CP028861.1, DQ147793.1, EF488989.1, KY312011.1; MW301936.1: *I. ricinus*, France). The next 29 sequences showed 88.80–99.10% similarity to *B. afzelii* (GenBank: CP009058.1, CP018262.1, MW301927.1), while 15 were 93.29–98.93% identical to *B. burgdorferi* s.s. (GenBank: CP031412.1; CP002228.1 and CP017201.1: humans, USA). *Borrelia valaisiana* was identified in nine samples, with similarity ranging from 91% to 98.24% to *Ixodes* ticks from France and Russia (GenBank: MW301935.1, CP009117.1). One sequence was 97.05% identical to *B. lusitaniae* found in *I. ricinus* in Turkey (GenBank: AB091820.1), and finally, six sequences were 96.83–98.94% similar to *B. miyamotoi* (GenBank: CP046389.1). One *Borrelia*-positive *D. reticulatus* female was identified as *B. garinii* with 89.43% similarity (GenBank: CP028861.1). Sequencing analysis for *Babesia*-positive samples (three male *D. reticulatus*) identified all of them as *B. canis* with 89.50–96.83% similarity (GenBank: MN704759.1, MK934420.1).

Sequencing of samples positive for *A. phagocytophilum* showed 100% similarity with the *A. phagocytophilum* strain Webster (188/188 bp) (GenBank: NR_044762.1).

### 3.2. Co-Infections

Overall, simultaneous presence of two different pathogens was detected in 14 *I. ricinus* ticks (1.7%; 14/842), among which 13 were collected in Białystok (2.8%; 13/460). The most prevalent co-infection was *B. afzelii* and *B. microti*, confirmed in 10 specimens (6 females, 3 males, 1 nymph). The remaining three samples, all females, were coinfected with *B. afzelii* and *B. venatorum*, *B. burgdorferi* s.s. and *B. microti*, as well as *B. garinii* and *B. microti*, respectively. Presence of *B. afzelii* and *A. phagocytophilum* was confirmed in one male tick from Augustów.

### 3.3. Phylogenetic Analysis

**Figure 4:**
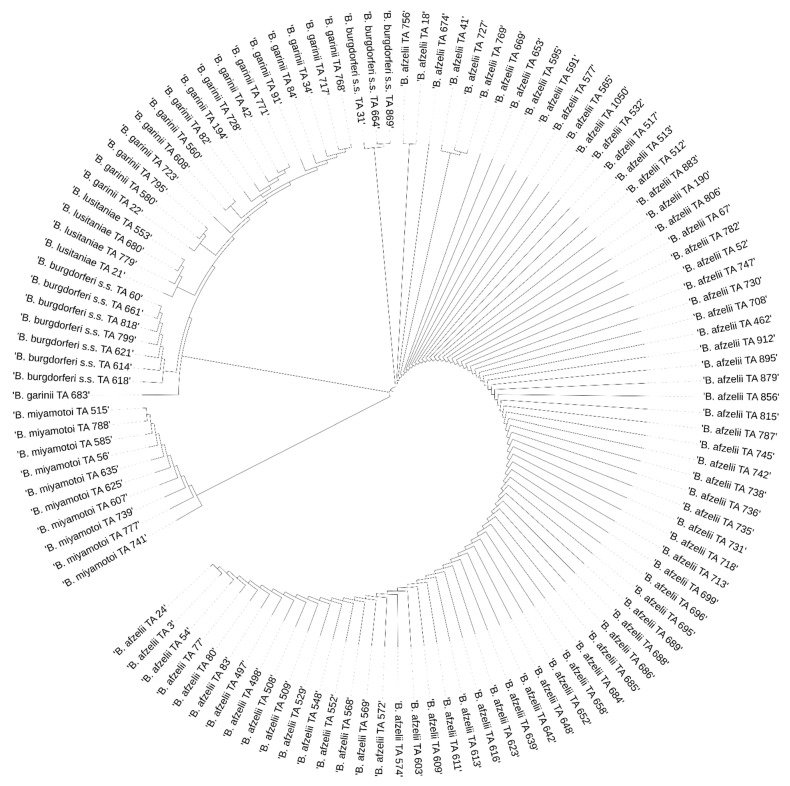
Phylogenetic analysis of the *Borrelia* spp. sequences obtained from *Ixodes ricinus* in Białystok.

**Figure 5:**
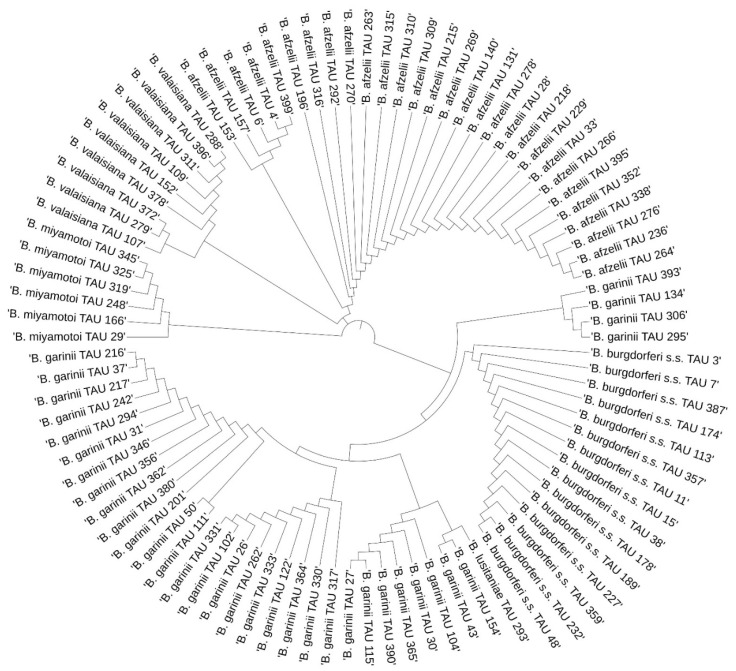
Phylogenetic analysis of the *Borrelia* spp. sequences obtained from *Ixodes ricinus* in Augustów.

**Figure 6:**
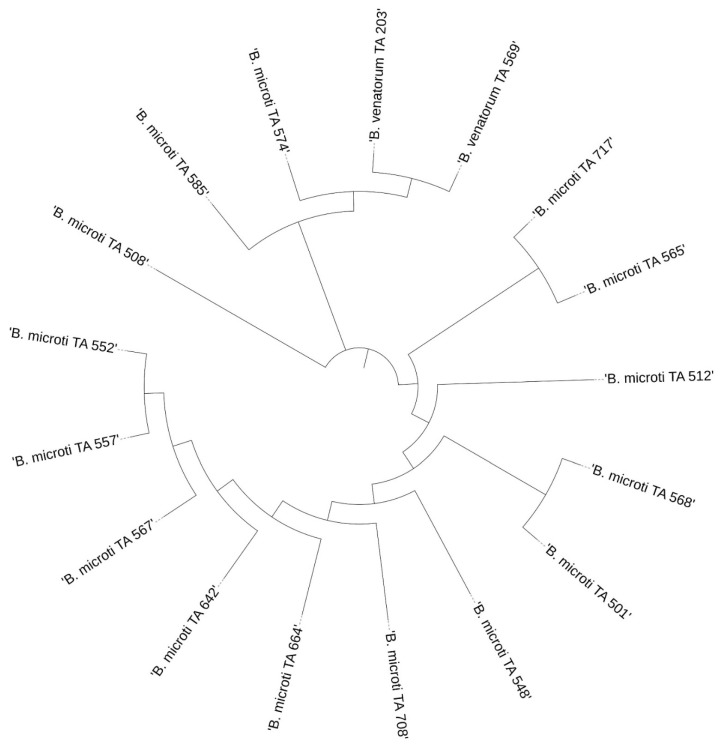
Phylogenetic analysis of the *Babesia* spp. sequences obtained from *I. ricinus*.

**Figure 7:**
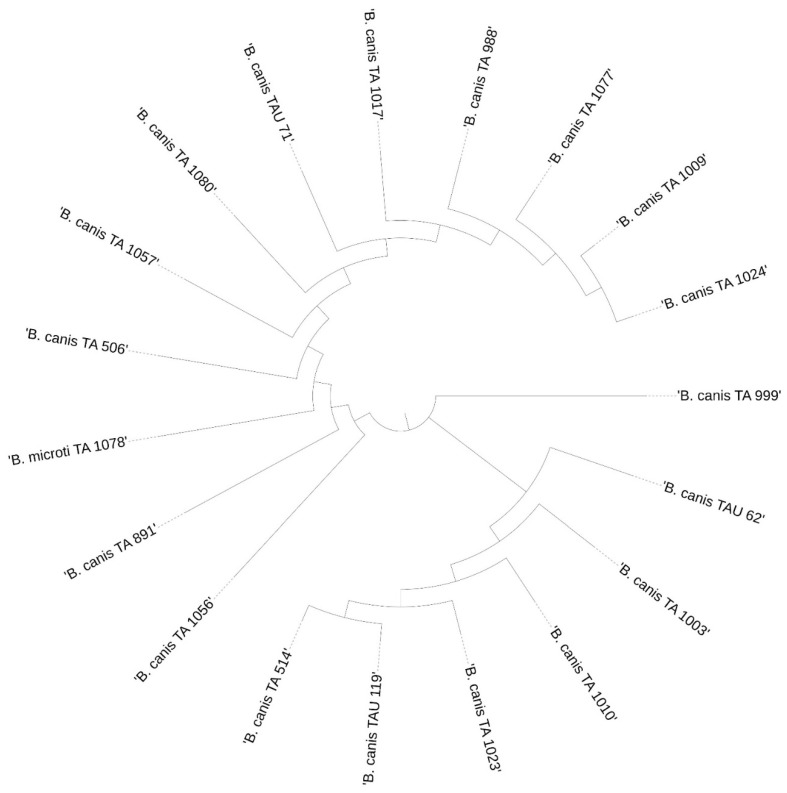
Phylogenetic analysis of the *Babesia* spp. sequences obtained from *D. reticulatus*.

The results of the phylogenetic analysis are presented in a graphical form in Figure 4, Figure 5, Figure 6 and Figure 7.

### 3.4. Statistical Analysis

**Figure 8:**
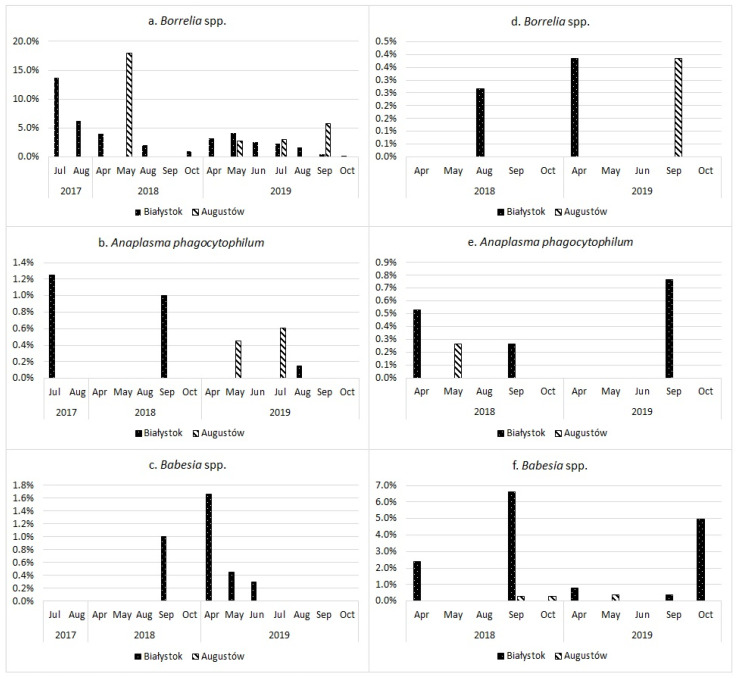
Prevalence of *Borrelia* spp., *Babesia* spp., and *Anaplasma phagocytophilum* in collected *Ixodes ricinus* (**a**–**c**) and *Dermacentor reticulatus* (**d**–**f**) ticks, divided by the sampling month and site.

Statistical analysis revealed significant differences in several categories. Data from previous and present research used in the evaluation is presented in Figure 8.

#### 3.4.1. Mann-Whitney Test

Statistically significant results were obtained in the following categories (Table 3).

##### Overall Infection Rate

Comparative analysis revealed a statistically significant difference between *Borrelia* spp. infection rate in *I. ricinus* and *D. reticulatus*, with higher prevalence in *I. ricinus* ticks. Moreover, significantly more *D. reticulatus* ticks were infected with *Babesia* spp.

##### Air Temperature

For April–July, a statistically significant difference was confirmed in *Borrelia* spp. infection rates in ticks collected in over 20 °C temperature. Opposite results were obtained for *Babesia* spp. The same relations in both pathogens were observed in August–October.

##### Relative Air Humidity

The comparative analysis revealed a higher amount of *Babesia* spp. infections in ticks collected during periods of air relative humidity below 80% humidity in April–July. In contradiction, *Borrelia* spp. was found more frequently in ticks sampled in over 80% humidity in August–October.

##### Sampling Season

Comparative analysis of infection rates in individual developmental stages in relation to the sampling season revealed statistically significant differences for *Borrelia* spp. It was established that more adults were positive for this pathogen if collected in April–July, while higher prevalence was noticed for nymphs sampled in August–October.

#### 3.4.2. Multivariate Logistic Regression Model

Multivariate logistic regression analysis showed that, for *Borrelia* spp. infections, D. reticulatus ticks were 97.16 times less likely to be infected with this pathogen as compared to *I. ricinus*. Moreover, the chance of detecting *Borrelia* spp. increased by 1.35 times in the successive sampling years and decreased by 1.59 times in males and nymphs, as compared to females (Table 4).

## 4. Discussion

Up until the 1980s, reports on infections in ticks in urban landscapes were incidental. Ever since then, the number of publications on this subject rose, presumably due to rapid development of recreational areas and green tourism, as well as progressing global urbanization [2].

Sequencing analysis of *Borrelia*-positive *I. ricinus* ticks identified the majority as *B. afzelii* and *B. garinii*, both in ticks collected in Białystok and Augustów. Those two *Borrelia* species are predominant in Europe [25], which was confirmed in other studies, including those from urban areas [6,7,26,27,28]. In this study, *B. lusitaniae* was confirmed in four *I. ricinus* ticks. Other than the current study, its presence was detected in two studies focusing on urban surroundings [7,27].

Interestingly, 6.4–8.4% of *I. ricinus* ticks were positive for *B. miyamotoi*, the causative agent of relapsing fever. Literature data regarding this spirochete presence in urban surrounding is scarce. However, *B. miyamotoi* was detected in such studies in Poland (4.7%) [26] and Switzerland (4.2%) [7]. Krause et al. suggests that *B. miyamotoi* may be prevalent in endemic borreliosis areas [29]. Human cases of *B. miyamotoi* infection were first reported in Russia in 2011 [30] and were since then described in multiple studies across Europe, the USA, and Japan [29,31,32].

It is worth emphasizing once again that Lyme disease incidence in the study region (107.7 per 100,000 people) is twice as high as the average in Poland (53.7 per 100,000 people) [14]. It is also worth noting that overall *B. burgdorferi* s.l. infection rates (23.5% and 23.0% for Białystok and Augustów, respectively) obtained in this study were also higher than the mean prevalence of *B. burgdorferi* s.l. in *I. ricinus* ticks in Europe (12.3%). Strnad et al. highlight that infection rates appear to increase significantly from western to eastern Europe [33].

*B. afzelii* was detected in only two *D. reticulatus* ticks (0.7%). This spirochete was also identified in other studies from Poland, although only in those from rural areas (0.09–1.6%) [34,35,36]. Low prevalence of *B. burgdorferi* s.l. in *D. reticulatus* ticks was confirmed in multiple studies in Europe [37,38,39,40], which may suggest that *D. reticulatus* ticks are ineffective vectors for this pathogen. In their study, Rudolf et al. examined the effect of *D. reticulatus* salivary glands and midgut extract on the growth, motility, and morphology of *B. garinii* in vitro. It was revealed that the extracts inhibited the growth of the spirochete [41].

The statistical analysis revealed higher median *Borrelia* spp. infection rate in ticks collected in temperatures above 20 °C in both seasons. It is known that the questing activity of *I. ricinus* nymphs and adults ranges from March to October [42], with a peak in AprilMay [43]. In a previous study that collected data on *I. ricinus* ticks from urban areas in Europe, it was revealed that temperature over 20 °C was connected to greater *B. burgdorferi* s.l. prevalence [44]. A relationship between higher mean temperatures and an increase in Lyme disease incidence was also observed by other studies [45,46]. As Keith et al. note, this may be further connected to the increase of human recreational activity in the warmer weather, thus higher tick exposure [45]. *Babesia* spp. infections were detected more frequently in temperatures below 20 °C and <80% humidity. In this study, *D. reticulatus* ticks were found to be primarily infected with *Babesia* spp. Additionally, all of the specimens were adults, who are most active during early spring (March-April) and autumn (September-October) [47], which, in Poland, are associated with lower temperatures. In comparison to *I. ricinus* ticks, *D. reticulatus* show higher resilience to colder environmental conditions [47,48].

In this study, the presence of *A. phagocytophilum* was confirmed in 0.7–1.8% *I. ricinus* ticks. Similar prevalence was reported in other Polish cities (1.7–3%) [11,49]. In Europe, *A. phagocytophilum* was detected in urban areas in Germany (1.7–3.8%) [10,27,50], Ukraine (5.2%) [5], Czech Republic (0–5.2%) [4,6], Switzerland (1.4%) [8], and Slovakia (3.1–7.2%) [51]. In a comprehensive study conducted by Derdakova et al. in various habitats across Slovakia, the Czech Republic, and Austria, it was found that the mean prevalence of *A. phagocytophilum* was 3.8% [52].

Overall, 1.1% of *D. reticulatus* ticks were infected with *A. phagocytophilum* in the current study, which is consistent with previous findings [15]. Similar results were obtained in Kyiv, Ukraine (0–1%) [5,53], while in the outskirts of Berlin, Germany, none of the collected *D. reticulatus* ticks were positive for this pathogen [39]. Comparable values were reported in studies conducted in rural areas of Poland and Serbia (0–1.1% and 1.9%, respectively) [36,40,54]. Results obtained in the current study most likely reflect the availability and population density of *A. phagocytophilum* hosts, such as rodents, hedgehogs, ungulates, foxes, and birds [5,11,51], which are necessary for the completion of the *A. phagocytophilum* life cycle, since this bacterium is not transmitted transovarially [55].The majority of *Babesia* in *I. ricinus* ticks were identified as *B. microti* in this study. Similar results were obtained by Wójcik-Fatla et al. in their research on recreational sites of eastern Poland [56]. It is worth noting that other studies focused on urban areas identified *B. venatorum* as the most prevalent [7,49,57]. Interestingly, one of the identified *B. microti* sequences (KP055650.1) is 100% identical to the pathogenic Jena/Germany strain. However, as stressed by Obiegala et al., it does not mean that the newly detected sequence is also pathogenic [58].

*B. canis* was the predominant pathogen identified in *D. reticulatus* ticks in this study (6.7% of all specimens), similar to the previous study (6.8%) [15]. Comparable results (4.18%) were obtained by Mierzejewska et al., who studied *D. reticulatus* ticks collected from multiple localities in eastern, central, and western Poland. In that study, *B. canis* was found only in ticks collected in the Eastern part of the country [34]. Noteworthy, Eastern Poland belongs to the European macro-region for *D. reticulatus* presence [59]. It is reflected by reported canine babesiosis cases. In a study conducted by Dwużnik et al., the authors collected data from 42 veterinary clinics from Eastern and Western Poland and reported 1558 cases of canine babesiosis. Interestingly, the majority (1532) of them came from clinics in the Eastern part of the country [60]. *B. canis* was also detected in a number of different studies on *D. reticulatus* ticks from Ukraine, Latvia, Lithuania, Slovakia, and Poland (0.63–3.4%) [53,56,61,62,63]. Notably, two studies from Poland and Serbia reported exceptionally high *B. canis* prevalence (21.3% and 20.8%, respectively) [35,40].

In this study, one *D. reticulatus* was infected with *B. microti* (0.4%), which is consistent with previous findings (0.8%) [15]. Other studies from Poland report 0.04–4.5% infection rate [34,36,54,64]. It is worth noting that the detected sequence was the same potentially pathogenic sequence as described in *I. ricinus*. Although it is known that *D. reticulatus* ticks rarely feed on humans [65], they significantly contribute to the circulation of pathogens, including those potentially harmful to humans, in the environment.

No *I. ricinus* ticks tested positively for *C. burnetii*, the causative agent of Q fever. Similar values (0–0.2%) were reported in studies conducted in Poland [66,67], Switzerland [68], Austria [69], and Sweden [70]. A notably higher infection rate (15.9%) was obtained by Szymańska-Czerwińska et al. in *I. ricinus* ticks collected from forests in south-eastern Poland [71]. In other European countries, obtained prevalence in rural areas was 0–4.9% in Slovakia [72,73,74], 1.7% in Belarus [38], and 1.9% in Germany [75].

Similarly, *C. burnetii* was not detected in any of the tested *D. reticulatus* ticks, which is consistent with results obtained in other studies [38,67,72,76]. Low prevalence of *C. burnetii* was detected in studies from Slovakia (2.1%) [74] and Serbia (3.7%) [40].

*Bartonella* spp. was not detected in any *I. ricinus* ticks investigated in this study. Similar values were reported in other European studies, both in urban Germany [77,78] and rural areas [38,40,79]. This pathogen has been reported in other research conducted in Poland with 1.7–4.8% prevalence, although all infected ticks were collected either from vegetation in rural areas or from animals [63,80,81]. In the current study, no *D. reticulatus* ticks were infected with *Bartonella* spp. Comparable results were obtained in research focused on urban areas: 0.5% in Warsaw, Poland [80] and 1.0% in Kyiv, Ukraine [53], as well as in rural surroundings: 0.6% in Belarus [38] and 0% in Serbia [40].

In this study, no *I. ricinus* tested positively for *Rickettsia* spp. In comparison, the presence of this pathogen was confirmed in urban parks in Warsaw (2.9–7.7%) [11,66]. In other European countries, infection rates reported in ticks from city landscapes were also higher, ranging from 5.7% to 16.5% [4,7,8,53,82,83]. Exceptionally high prevalence was confirmed within urban areas of Hanover and Hamburg, Germany (50.8% and 52.5%, respectively) [10,84].

Interestingly, also no *D. reticulatus* ticks were found to be infected with *Rickettsia* spp. in the current study. This pathogen has been reported with a high prevalence rate (40.7–56.7%) in natural sites in Poland [34,36,66,85,86,87]. Studies from urban areas in Kyiv, Ukraine, revealed 10.1–35.7% infection rate [5,53]. In other European countries, reported infection rates in natural sites were similar (14–21.4%) [65,88]. Given such discrepancies between *Rickettsia* spp. prevalence obtained in this and other studies, further research in the study area, focusing on this pathogen, is needed.

## 5. Conclusions

In conclusion, the molecular investigation carried out in this study confirms that *I. ricinus* and *D. reticulatus* ticks present within urban areas of the northeastern Poland are infected with at least three pathogens: *Borrelia* spp., *A. phagocytophilum*, and *Babesia* spp. Moreover, results reveal that the prevalence of *B. burgdorferi* s.l. is equal or even higher than in natural ecosystems. As this is the first study on ticks in cities of northeastern Poland, it provides valuable information for tick-borne pathogen surveillance.

## Figures and Tables

**Table 1:** The number of *Ixodes ricinus* and *Dermacentor reticulatus* ticks collected from the study areas.

		*Ixodes ricinus*						*Dermacentor reticulatus*		
Collection Site	Sampling Year	Females	Males	Nymphs	Larvae	Total	Females	Males	Nymphs	Total
Białystok	2017	33	41	6	-	**80**	-	-	-	**-**
	2018	16	14	2	-	**32**	-	-	-	**-**
	2019	127	92	79	50	**348**	150	94	8	**252**
	**Total**	**176**	**147**	**87**	**50**	**460**	**150**	**94**	**8**	**252**
Augustów										
“Królowa Woda” resort	2018	18	15	35	-	**68**	5	4	-	**9**
Public Beach		-	-	-	-	**-**	-	-	-	**-**
“Królowa Woda” resort	2019	3	14	14	-	**31**	1	-	-	**1**
Public Beach		42	31	183	27	**283**	6	2	-	**8**
	**Total**	**63**	**60**	**232**	**27**	**382**	**12**	**6**	**-**	**18**

**Table 2:** The percentage and number of pathogens detected in *Ixodes ricinus* and *Dermacentor reticulatus* ticks collected during the study.

	*Ixodes ricinus*					*Dermacentor reticulatus*			
Collection Site	Sampling Year	Bor	Bab	Ap	Total	Bor	Bab	Ap	Total
Białystok	2017	20% (16/80)	0% (0/80)	1.3% (1/80)	**21.3%** **(17/80)**	-	-	-	**-**
	2018	21.9% (7/32)	3.1% (1/32)	3.1% (1/32)	**28.1%** **(9/32)**	-	-	-	**-**
	2019	27.3% (95/348)	4.6% (16/348)	0.3% (1/348)	**28.4% (99/348)**	0.4% (1/252)	6.4% (16/252)	0.8% (2/252)	**7.6%** **(19/252)**
	**Total**	**25.7% (118/460)**	**3.7%** **(17/460)**	**0.7%** **(3/460)**	**27.2% (125/460)**	**0.4%** **(1/252)**	**6.4%** **(16/252)**	**0.8%** **(2/252)**	**7.6%** **(19/252)**
Augustów									
“Królowa Woda” resort	2018	26.5% (18/68)	0% (0/68)	0% (0/68)	**26.5%** **(18/68)**	0% (0/9)	22.2% (2/9)	11.1% (1/9)	33.3% (3/9)
“Królowa Woda” resort	2019	12.9% (4/31)	0% (0/31)	3.2% (1/31)	**16.1%** **(5/31)**	0% (0/1)	0% (0/1)	0% (0/1)	0% (0/1)
Public Beach		25.4% (72/283)	0% (0/283)	2.1% (6/283)	**27.0% (77/283)**	12.5% (1/8)	12.5% (1/8)	0% (0/8)	25% (2/8)
	**Total**	**24.6% (94/382)**	**0%** **(0/382)**	**1.8%** **(7/382)**	**26.2% (100/382)**	**5.5%** **(1/18)**	**16.7%** **(3/18)**	**5.5%** **(1/18)**	**27.7%** **(5/18)**

Bor—Borrelia spp., Bab—Babesia spp., Ap—Anaplasma phagocytophilum.

**Table 3:** Comparison of tick-borne pathogen infection rates in *Ixodes ricinus* and *Dermacentor reticulatus* ticks, according to seasonal variety, air temperature, and relative air humidity.

Category	Variable	*p* Value
Overall infection rate between *Dermacentor reticulatus* and *Ixodes ricinus*	*Borrelia* spp.	0.001
	*Babesia* spp.	0.001
Temperature in April-July	*Borrelia* spp.	0.003
	*Babesia* spp.	0.001
Temperature in August-October	*Borrelia* spp.	0.001
	*Babesia* spp.	0.001
Relative air humidity in April-July	*Babesia* spp.	0.001
Relative air humidity in August-October	*Borrelia* spp.	0.001
Sampling season (April-July, August-October)	*Borrelia* spp. in females	0.001
	*Borrelia* spp. in males	0.001
	*Borrelia* spp. in nymphs	0.046

**Table 4:** Multivariate logistic regression model for *Borrelia* spp. infection rate.

Parameter	Odds Ratio	*p* Value	95% Confidence Interval	
Tick species	0.01	0.001	0.003	0.033
Sampling year	1.35	0.024	1.04	1.76
Developmental stage	0.63	0.001	0.53	0.75

## Data Availability

The data sets used and/or analyzed during the current study are available from the corresponding author on request.
